# Comparative effectiveness of dexmedetomidine and meperidine for the treatment of postanesthetic shivering: a propensity score-matched retrospective cohort study

**DOI:** 10.1186/s12871-026-04047-9

**Published:** 2026-06-26

**Authors:** Ji Wook Kim, Sumi Kim, Kichul Lee, Jaeuk Na, Hyung Joo Chung, Ju Deok Kim, Donghee Kang

**Affiliations:** 1 Department of Anesthesiology and Pain Medicine, Kosin University College of Medicine, 262 Gamcheon-ro, Seo-gu, Busan, 49267 Republic of Korea; 2 Department of Pathology, Veterans Health Service Busan Hospital, 420 Baegyang-daero, Sasang-gu, Busan, Korea

**Keywords:** Dexmedetomidine, Meperidine, Post-anesthesia care unit, Propensity score, Retrospective studies, Shivering

## Abstract

**Background:**

Postanesthetic shivering (PAS) is a common and distressing complication in the post-anesthesia care unit (PACU). Meperidine has traditionally been used for PAS treatment, but interest in non-opioid alternatives has increased. We compared the effectiveness and safety of dexmedetomidine versus meperidine for the treatment of PAS.

**Methods:**

This retrospective cohort study included 484 adult patients who developed PAS in the PACU after surgery between January and December 2024 and received intravenous dexmedetomidine or meperidine as the initial treatment. The primary outcome was treatment failure, defined as the need for rescue anti-shivering medication before PACU discharge. Secondary outcomes included hypotension, bradycardia, postoperative nausea and vomiting, clinically significant respiratory depression, PACU length of stay, and PACU fentanyl consumption. After 1:1 propensity score matching, 149 matched pairs were analyzed.

**Results:**

In the matched cohort, dexmedetomidine was associated with a lower rate of treatment failure than meperidine (3.4% vs. 10.1%; OR 0.33, 95% CI 0.12–0.92; *p* = 0.033). Hypotension was more frequent in the dexmedetomidine group (6.7% vs. 1.3%; OR 5.00, 95% CI 1.10–22.82; *p* = 0.038). No clear between-group differences were found in bradycardia, postoperative nausea and vomiting, PACU length of stay, or fentanyl consumption, and no documented clinically significant respiratory depression occurred in either group. Sensitivity analyses showed similar results for the primary outcome.

**Conclusion:**

In patients with PAS in the PACU, dexmedetomidine was associated with a lower risk of treatment failure than meperidine, but a higher risk of hypotension. Dexmedetomidine may be an effective alternative when appropriate hemodynamic monitoring is available.

**Clinical trial registration:**

Not applicable.

**Supplementary Information:**

The online version contains supplementary material available at https://doi.org/10.1186/s12871-026-04047-9.

## Introduction

Postanesthetic shivering (PAS) is a common and distressing complication during recovery from anesthesia. Beyond patient discomfort, PAS may increase metabolic demand and cardiovascular stress, which can be clinically relevant in vulnerable patients. Therefore, prompt and effective pharmacologic treatment of established PAS remains an important component of post-anesthesia care unit (PACU) care [1, 2].

Meperidine, an opioid, has traditionally been used as the standard pharmacologic treatment for PAS [2]. However, perioperative care has increasingly emphasized opioid-minimization strategies, particularly within enhanced recovery after surgery (ERAS) pathways [3–5]. In this context, interest has grown in non-opioid alternatives that can effectively treat PAS while avoiding additional opioid exposure. Among several candidate agents, dexmedetomidine has gained attention as a non-opioid agent that may effectively treat PAS by stabilizing sympathetic activity in addition to its sedative properties. Dexmedetomidine is a highly selective α2-adrenergic receptor agonist and is known to exert antishivering effects by acting on the hypothalamic thermoregulatory center, thereby lowering the vasoconstriction threshold and shivering threshold in a dose-dependent manner [6]. This central mechanism is understood not merely as inhibition of peripheral vasoconstriction but as a downward modulation of thermoregulatory thresholds [7], and it may be clinically advantageous when additional opioid exposure is undesirable [8]. Nevertheless, prior studies have predominantly investigated prophylactic dexmedetomidine administration for PAS prevention, whereas its therapeutic use after PAS onset has been less well studied [9, 10]. In particular, only a few studies have directly compared dexmedetomidine with meperidine in real-world clinical settings while assessing clinically meaningful outcomes such as the need for rescue medication and PACU recovery metrics.

We hypothesized that dexmedetomidine would be associated with a lower risk of treatment failure than meperidine while demonstrating an acceptable safety profile in patients who developed PAS in the PACU. Accordingly, we applied propensity score matching to a retrospective cohort to compare the effectiveness and safety of dexmedetomidine versus meperidine for PAS treatment.

## Methods

### Study design and participants

This study was a retrospective cohort study based on the electronic medical record (EMR) system. The study was approved by the Institutional Review Board (IRB) of Kosin University Gospel Hospital (KUGH 2025-10-003), and the requirement for informed consent was waived because the study involved retrospective data analysis with minimal risk to participants. The study was conducted in accordance with the ethical principles of the Declaration of Helsinki, and all data were anonymized prior to analysis.

We initially reviewed the medical records of 3,943 adult (≥ 19 years) patients who underwent surgery under general or regional anesthesia at our institution between January and December 2024. Patients who met any of the following exclusion criteria were excluded from the analysis: (1) American Society of Anesthesiologists (ASA) physical status class IV or higher, (2) a history of allergy or hypersensitivity to the study drugs (dexmedetomidine, meperidine), (3) hemodynamically unstable patients arriving at the PACU receiving continuous intravenous vasopressor infusion (at the time of PACU admission), (4) patients who remained intubated at the end of surgery or at the time of PACU discharge, (5) pregnant or breastfeeding patients, and (6) patients unable to reliably report symptoms due to cognitive impairment. Among patients who did not meet any exclusion criteria, those who developed PAS in the PACU and received intravenous dexmedetomidine or meperidine as the initial treatment were selected for the final analysis.

### Treatment protocol and outcome measures

In the PACU, the presence of shivering was assessed on arrival by PACU nurses and anesthesiologists. Thereafter, patients were routinely reassessed at 5-min intervals as part of standard PACU vital-sign monitoring, during which the presence of shivering and the overall clinical status were checked. When shivering was identified, its severity was graded using a 5-point scale (0–4): 0, no shivering; 1, piloerection or peripheral vasoconstriction; 2, muscular activity confined to one muscle group; 3, muscular activity observed in more than one muscle group (not generalized); and 4, gross muscular activity involving the whole body. Patients with clinically significant shivering, defined as a shivering grade of ≥ 2, received dexmedetomidine or meperidine according to the attending anesthesiologist’s clinical judgment. Dexmedetomidine was administered intravenously at 0.3–0.5 µg/kg over approximately 2 min, and meperidine was administered intravenously at 0.5 mg/kg. Following drug administration, the clinical response, including persistence or cessation of shivering and the need for rescue medication, was closely monitored at the bedside according to routine PACU practice. Rescue medication was administered when shivering persisted or did not improve sufficiently after the initial treatment, as determined by the attending anesthesiologist.

Demographic characteristics and perioperative information were retrospectively extracted from the EMR and PACU records. Collected variables included baseline characteristics (age, sex, comorbidities, ASA physical status class, type of surgery, and anesthetic technique) as well as PAS-related documentation in the PACU (administered drug and dose, symptom improvement, and additional drug administration), tympanic temperature and PACU length of stay, acetaminophen administration (before administration of PAS treatment drugs), hypotension or vasopressor administration, bradycardia or atropine administration, respiratory depression or airway/ventilatory intervention, and the occurrence of nausea and vomiting.

The primary outcome was treatment failure, defined as persistent or worsening PAS after administration of intravenous dexmedetomidine or meperidine as the initial treatment, requiring additional medication (rescue medication) before PACU discharge. To evaluate drug safety and recovery quality, we assessed hypotension in the PACU (systolic blood pressure < 90 mmHg or vasopressor administration), bradycardia (heart rate < 50 beats/min or atropine administration), postoperative nausea and vomiting (PONV), clinically significant respiratory depression, PACU length of stay, and PACU fentanyl consumption. Clinically significant respiratory depression was defined as any documented respiratory event after administration of the study drug and before PACU discharge that required active intervention beyond routine oxygen supplementation, including airway support, assisted ventilation, naloxone administration, reintubation, or delayed PACU discharge due to respiratory compromise.

### Sample size and propensity score matching

As this was a retrospective observational study, the sample size was determined by the available patient data during the study period. To minimize confounding by indication, treatment effects were compared in a 1:1 propensity score-matched cohort. Covariates were selected based on clinical relevance before propensity score matching, considering factors related to perioperative shivering risk, treatment response, treatment selection, and hemodynamic safety. Propensity scores were estimated using a logistic regression model with treatment group (dexmedetomidine vs. meperidine) as the dependent variable. Age, sex, type of surgery, anesthetic technique (general anesthesia vs. regional anesthesia), duration of anesthesia, and ASA physical status class were included to account for baseline demographic, surgical, anesthetic, and clinical characteristics. Comorbidities, including hypertension, diabetes mellitus, and cardiovascular disease, were included to account for baseline clinical status and potential susceptibility to hemodynamic adverse events. Tympanic temperature at PACU arrival was included because hypothermia is a major contributor to PAS, and pre-treatment acetaminophen administration was included because acetaminophen may have anti-shivering effects. Binary outcomes were reported as odds ratios with 95% confidence intervals and as absolute risk differences, whereas continuous outcomes were compared using mean differences.

Type of surgery was categorized into three levels (low/moderate/high) according to perioperative hypothermia risk based on surgical field exposure and body cavity exposure. The low-risk category was defined as superficial surgeries with no or minimal body cavity exposure (skin and soft tissue, breast, thyroid/parathyroid) and limited endoscopy-based procedures. The high-risk category was defined as surgeries with extensive body cavity exposure, including open abdominal surgery (major abdominal surgery involving the stomach, colon, or hepatobiliary-pancreatic systems) or thoracic surgery. Procedures with moderate exposure that did not meet these definitions (laparoscopic abdominal surgery, spine surgery, joint arthroplasty, head and neck surgery, arthroscopy) were classified as moderate risk. Type of surgery and ASA physical status class were modeled as categorical variables to avoid imposing a linear trend across categories.

Matching was performed using nearest-neighbor matching with a caliper width of 0.2 times the standard deviation of the logit of the propensity score. Covariate balance after matching was assessed using the standardized mean difference (SMD), with SMD < 0.1 considered indicative of adequate balance. Missing data, if present, were handled using complete-case analysis. In the final analytic dataset, no missing values were observed for propensity score covariates or outcome variables; therefore, no additional exclusions resulted from the complete-case approach.

### Statistical analysis

Continuous variables were presented as mean ± standard deviation, and categorical variables were presented as number (%). Accounting for the matched pairs generated by 1:1 propensity score matching, binary outcomes (treatment failure, hypotension, bradycardia, PONV) were compared using a conditional logistic regression model, and odds ratio (OR) with 95% confidence intervals (CI) were estimated. Because no clinically significant respiratory depression occurred in either group, this outcome was summarized descriptively and no effect estimate was calculated. In addition, for each binary outcome, the absolute risk difference (RD; percentage points) between the dexmedetomidine and meperidine groups was calculated and reported. Continuous outcomes (PACU length of stay and fentanyl consumption) were analyzed using a linear mixed model including matched pairs as a random effect, and the between-group mean difference (MD) with 95% CI was presented.

To assess the robustness of the primary analysis, two prespecified sensitivity analyses were performed. First, 1:1 matching was repeated using a more stringent caliper width (0.1 times the standard deviation of the logit of the propensity score), and the same analytical approach was applied. Second, in the unmatched full cohort, we performed multivariable regression analyses to estimate adjusted odds ratios for binary outcomes and adjusted mean differences for continuous outcomes.

All tests were two-sided, and *p* < 0.05 was considered statistically significant. Statistical analyses were performed using R (R Foundation for Statistical Computing, Vienna, Austria) version 4.5.2.

## Results

**Fig. 1:**
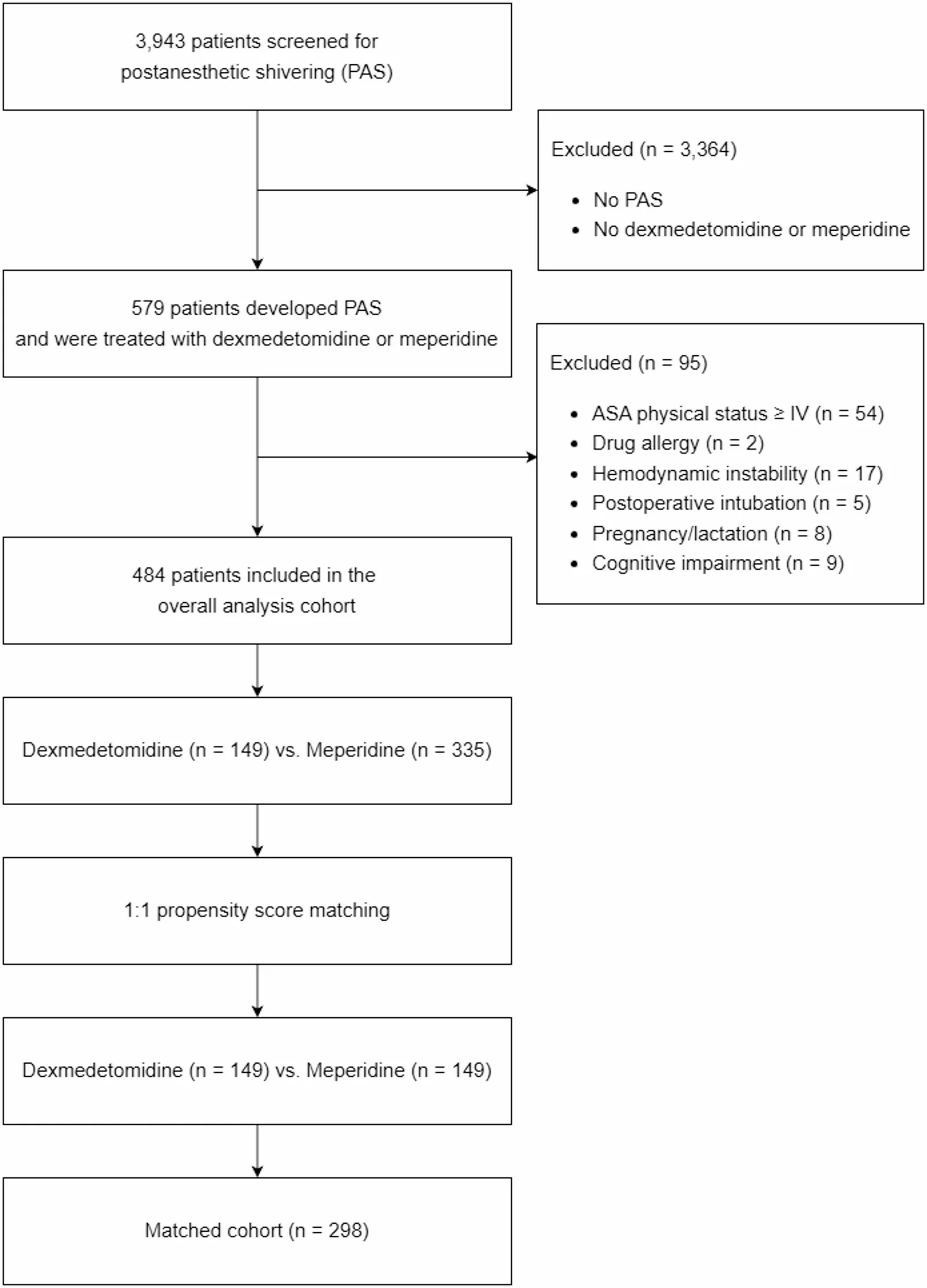
Flow diagram

Among 3,943 patients screened, PAS occurred in 579 patients (14.7%) who received dexmedetomidine or meperidine. After excluding 95 patients who met the exclusion criteria, 484 patients were included in the full analytic cohort. After 1:1 propensity score matching, 149 pairs (total 298 patients) were included in the final analysis, and the participant selection and matching process was presented in Fig. 1.

Baseline demographic and surgical characteristics before and after matching were presented in Table 1. The SMDs in Table 1 were presented as absolute values. After matching, baseline characteristics were generally well balanced, although residual imbalance remained in two categories of type of surgery. The SMDs for the high- and moderate-risk hypothermia categories were 0.144 and 0.143, respectively, both exceeding 0.1. Clinical outcomes were presented in Table 2. In the unmatched full cohort (*n* = 484), the dexmedetomidine group had a significantly lower treatment failure rate than the meperidine group (3.4% vs. 9.0%, OR 0.35, 95% CI 0.13–0.93, *p* = 0.035; Supplementary Table 1). After matching, dexmedetomidine remained associated with a significantly lower risk of treatment failure than meperidine (3.4% vs. 10.1%; OR 0.33, 95% CI 0.12–0.92; *p* = 0.033). The absolute risk difference was − 6.7% points. 

**Table 1:** Baseline characteristics before and after propensity score matching

	Before matching			After matching		
	Meperidine (*n* = 335)	Dexmedetomidine (*n* = 149)	SMD	Meperidine (*n* = 149)	Dexmedetomidine (*n* = 149)	SMD
Age (yr)	60.10 ± 14.40	58.19 ± 14.82	0.129	58.93 ± 15.48	58.19 ± 14.82	0.050
Sex (M/F)	140/195	59/90	0.045	60/89	59/90	0.014
Hypothermia risk (%)						
High	46 (13.7)	18 (12.1)	0.051	25 (16.8)	18 (12.1)	0.144
Moderate	113 (33.7)	49 (32.9)	0.018	39 (26.2)	49 (32.9)	0.143
Low	176 (52.5)	82 (55.0)	0.050	85 (57.0)	82 (55.0)	0.041
Anesthesia type (%)			0.021			0.075
General anesthesia	325 (97.0)	144 (96.6)		142 (95.3)	144 (96.6)	
Regional anesthesia	10 (3.0)	5 (3.4)		7 (4.7)	5 (3.4)	
ASA-PS (%)			0.178			0.085
ASA I	17 (5.1)	10 (6.7)		9 (6.0)	10 (6.7)	
ASA II	244 (72.8)	116 (77.9)		121 (81.2)	116 (77.9)	
ASA III	74 (22.1)	23 (15.4)		19 (12.8)	23 (15.4)	
Hypertension (%)	137 (40.9)	56 (37.6)	0.068	59 (39.6)	56 (37.6)	0.042
Diabetes mellitus (%)	96 (28.7)	37 (24.8)	0.089	33 (22.1)	37 (24.8)	0.062
Cardiovascular disease (%)	55 (16.4)	23 (15.4)	0.027	21 (14.1)	23 (15.4)	0.037
Anesthesia time (min)	211.10 ± 120.66	210.17 ± 114.75	0.008	200.57 ± 117.75	210.17 ± 114.75	0.084
Tympanic temperature (°C)	35.84 ± 0.20	35.88 ± 0.21	0.182	35.87 ± 0.21	35.88 ± 0.21	0.038
Acetaminophen use (%)	277 (82.7)	121 (81.2)	0.038	124 (83.2)	121 (81.2)	0.052

Data are presented as mean ± standard deviation or number of patients (%). SMD < 0.1 was considered indicative of adequate balance. *ASA-PS *American Society of Anesthesiologists physical status, *SMD* standardized mean difference

**Table 2:** Clinical outcomes in the propensity score-matched cohort (*n* = 149 pairs)

	Meperidine (*n* = 149)	Dexmedetomidine (*n* = 149)	Effect size (95% CI)	*p*-value	RD (%)
Primary outcome					
Treatment failure*	15 (10.1)	5 (3.4)	OR 0.33 (0.12, 0.92)	0.033	-6.71
Secondary outcomes					
Hypotension	2 (1.3)	10 (6.7)	OR 5.00 (1.10, 22.82)	0.038	+ 5.37
Bradycardia	5 (3.4)	8 (5.4)	OR 1.60 (0.52, 4.89)	0.410	+ 2.01
PONV	3 (2.0)	2 (1.3)	OR 0.67 (0.11, 3.99)	0.657	-0.67
Respiratory depression	0 (0.0)	0 (0.0)	Not estimable	-	0.00
Stay in PACU (min)	29.13 ± 13.26	26.88 ± 11.40	MD − 2.25 (–4.99, 0.50)	0.108	–
Fentanyl dose (µg)	59.56 ± 33.59	66.44 ± 34.85	MD 6.88 (–0.87, 14.62)	0.082	–

Values are presented as number of patients (%) or mean ± standard deviation. Effect sizes are presented as odds ratios for binary outcomes and mean differences for continuous outcomes, each with 95% confidence intervals. RD was calculated for binary outcomes as Dexmedetomidine − Meperidine and is expressed in percentage points. Treatment failure was defined as the need for additional rescue anti-shivering medication in the post-anesthesia care unit after initial administration of the study drug. The effect size for respiratory depression was not estimated because no events occurred in either group. *RD* risk difference, *OR* odds ratio, *MD* mean difference, *PONV* postoperative nausea and vomiting, *PACU* post-anesthesia care unit

With respect to safety outcomes, the incidence of hypotension was significantly higher in the dexmedetomidine group (6.7%) than in the meperidine group (1.3%) (OR 5.00, 95% CI 1.10–22.82, *p* = 0.038). No significant between-group differences were observed in the incidence of bradycardia or PONV, and neither PACU length of stay nor fentanyl consumption differed significantly between groups. No documented clinically significant respiratory depression occurred in either group after administration of the study drug and before PACU discharge.

Additional sensitivity analyses were performed (Supplementary Table 2), and the estimates for treatment failure showed a consistent direction across analytic conditions.

## Discussion

In this propensity score-matched cohort study, dexmedetomidine was associated with a lower proportion of patients requiring additional rescue medication for the treatment of PAS in the PACU compared with meperidine (3.4% vs. 10.1%). Although residual covariate imbalance remained in the high- and moderate-risk surgical categories classified according to hypothermia risk after matching, tympanic temperature at PACU arrival was included in the propensity score model to account for thermal status before PAS treatment. In sensitivity analyses addressing this residual imbalance, the direction of the association was maintained, and the effect estimates for the primary outcome were similar to those of the main analysis (Supplementary Table 2). These findings suggest that the association between treatment group and treatment failure was consistent across analytic approaches.

In the existing literature, studies evaluating the association between dexmedetomidine and PAS have largely focused on prophylactic administration, often within specific surgical populations and controlled research settings, using incidence or severity reduction as primary endpoints [9, 11, 12]. However, reductions in PAS incidence or severity reported in prophylactic studies do not directly reflect therapeutic effectiveness in patients who require treatment after PAS has already occurred. Therefore, evidence evaluating treatment effectiveness after the onset of PAS in the PACU is needed. Although a limited number of studies have directly compared dexmedetomidine and meperidine for PAS treatment, most were small, single-center randomized controlled trials conducted under controlled conditions [13]. In contrast, studies that quantify the effectiveness–safety balance in heterogeneous surgical populations under routine clinical practice remain limited. The need for additional rescue medication implies persistent patient discomfort attributable to PAS. It also necessitates further drug administration and observation, thereby serving as a clinically meaningful marker of additional treatment burden in the PACU. By defining this as the primary outcome, our study directly reflected the point at which clinical decision-making occurs in the PACU. Moreover, our evaluation of both treatment effectiveness and hemodynamic risk across diverse surgical cohorts complements the existing, largely efficacy-focused evidence base.

In this study, the dexmedetomidine dose used for PAS treatment was 0.3–0.5 µg/kg. This was substantially lower than the 0.5–1.0 µg/kg doses used in prior randomized controlled trials for PAS prophylaxis, in which dose-dependent adverse effects such as prolonged extubation time, sedation on PACU arrival, and increased atropine use were reported [11, 14–16]. In contrast, doses for treatment have been reported to be lower than prophylactic doses. Abdel-Ghaffar et al. reported that dexmedetomidine 0.3 µg/kg achieved a treatment response comparable to that of 0.5 µg/kg in the treatment of established PAS after lower abdominal surgery under spinal anesthesia, with fewer hemodynamic adverse events and less sedation in the 0.3 µg/kg group [13]. Yang et al. estimated the ED95 for treating severe shivering after cesarean delivery to be 0.39 µg/kg, and Lim Fern et al. similarly reported both therapeutic effectiveness and hemodynamic adverse events using 0.5 µg/kg in patients with grade 3–4 shivering [17, 18]. Notably, despite the considerably lower dosing range in our study than the 0.5–1.0 µg/kg range for which prophylactic efficacy has been demonstrated, the treatment failure rate remained low at 3.4%. This is consistent with the dose-finding results reported by Abdel-Ghaffar et al. and the ED95 estimated by Yang et al., and suggests that PAS control may be achievable with relatively low doses of dexmedetomidine [13, 17]. Prevention and treatment have different goals, and the degree of thermoregulatory modulation required may not be identical; at the time shivering occurs and treatment is required, symptom cessation could be achievable through a shift in the thermoregulatory threshold with comparatively smaller doses. This interpretation should be considered in the context of our study population, which excluded critically ill patients who arrived in the PACU on continuous vasopressors or remained intubated. Accordingly, the feasibility of PAS control with relatively low-dose dexmedetomidine may be most applicable to clinically stable PACU patients with continuous monitoring.

From a safety perspective, the dexmedetomidine group had a significantly higher risk of hypotension than the meperidine group. This finding is consistent with the known hemodynamic properties of dexmedetomidine as an α2-adrenergic agonist that produces sympatholysis [19, 20]. Prior studies have also repeatedly reported increased hypotension with dexmedetomidine; however, heterogeneity in the incidence and effect size is observed across studies because of differences in the definition of hypotension, administered dose and infusion duration, and patient populations. In a meta-analysis of PAS treatment studies, dexmedetomidine was associated with a higher risk of hypotension than tramadol, suggesting a trade-off between shivering control and hemodynamic stability [21]. Abdel-Ghaffar et al. reported dose-related differences in hemodynamic adverse events in a dose-comparison study, suggesting the possibility of a dose-dependent response [13]. In our primary analysis, the OR for hypotension in the dexmedetomidine group was higher than that in the meperidine group (OR 5.00, 95% CI 1.10–22.82). However, the effect size and statistical significance varied to some extent across sensitivity analyses, although the direction of the association remained similar across analytic approaches, with dexmedetomidine associated with a higher risk of hypotension. No major cardiovascular events such as myocardial infarction or stroke were documented in either group during the PACU stay; nonetheless, the possibility that dexmedetomidine-associated hypotension may be clinically relevant cannot be entirely excluded, particularly in patients with underlying cardiovascular disease. Accordingly, while uncertainty remains regarding the exact magnitude of risk, the consistent direction suggests that dexmedetomidine use may warrant careful hemodynamic monitoring in the PACU.

The infusion duration of dexmedetomidine is one parameter that may be adjusted to mitigate hypotension risk. Across the literature, the infusion duration for dexmedetomidine administered for PAS treatment has been reported to vary from 2 to 10 min [13, 18, 22]. Rapid bolus administration has been associated with hemodynamic fluctuations, including transient hypertension, bradycardia, and subsequent hypotension, and gradual administration over a defined period has therefore been proposed [23, 24]. However, when PAS has already occurred and requires treatment, rapid symptom control is often necessary; accordingly, some PAS treatment studies infused doses ≤ 0.5 µg/kg over 2–3 min [13, 17]. In our study, infusion over approximately 2 min was used based on clinical judgment; a low treatment failure rate was observed, and hemodynamic responses remained within a clinically manageable range. Nevertheless, given the increased hypotension observed in our cohort, we cannot exclude the possibility that the infusion rate contributed to hemodynamic variability. Even so, the commonly suggested 10-min infusion may be difficult to implement in situations requiring prompt PAS treatment. In this clinical context, administering low-dose dexmedetomidine over approximately 2 min may be interpreted as a pragmatic option that considers both rapid onset of effect and hemodynamic safety in the PACU.

Bradycardia has been reported with widely varying incidence (0–20%) in studies of dexmedetomidine for PAS treatment [13, 17, 18, 22]. This variation may be attributable to differences in the definition of bradycardia across studies (HR < 50 or < 60/min, or a 20% decrease from baseline), as well as variability in dose and infusion duration. These findings suggest that dexmedetomidine-associated bradycardia risk may depend on dose and administration conditions rather than being uniformly present or absent. In our study, bradycardia showed a trend toward higher incidence in the dexmedetomidine group but was not statistically significant. Therefore, this non-significant result should not be interpreted as evidence of equivalent safety between the two agents; rather, it suggests that the risk of clinically significant bradycardia was limited within the therapeutic dosing range and administration patterns used in our cohort. In other words, with appropriate dose selection and infusion conditions, bradycardia may not be a major limiting factor for PAS treatment.

The incidence of PONV did not differ significantly between groups. In this cohort, frequent prophylactic administration of ramosetron and the predominant use of propofol for induction may have contributed to a lower overall risk of PONV. No clear differences were observed in PACU length of stay or fentanyl consumption, which suggests that the therapeutic doses used in this study may not have caused clinically meaningful residual sedation. In addition, no documented clinically significant respiratory depression occurred in either group after administration of the study drug. This finding is consistent with the relatively low doses used for PAS treatment in this cohort and suggests that both treatments were clinically tolerable in the selected PACU population. In a meta-analysis by Sin et al., dexmedetomidine did not significantly prolong PACU length of stay, and the incidence of residual sedation did not differ from that in control groups, consistent with our findings [25]. Regarding postoperative pain, dexmedetomidine exerts analgesic effects at the level of the spinal dorsal horn via α2-adrenergic receptor activity, and evidence suggests that it can reduce postoperative pain and opioid/analgesic consumption [15, 25]. Taken together, these findings suggest that dexmedetomidine may be a clinically viable alternative to meperidine for PAS treatment in the PACU, particularly when minimizing opioid exposure is desired.

Several limitations should be noted. First, as a retrospective observational study in which treatment selection was determined by clinicians, confounding by indication cannot be fully excluded. Although patients with ASA physical status ≥ IV or those receiving continuous vasopressor infusion on PACU arrival were excluded, variables such as hemodynamic status immediately before drug administration (borderline bradycardia/hypotension) and the risk of opioid-related adverse effects may not have been fully captured as covariates in the propensity score model. These factors may have contributed to residual confounding affecting the estimated treatment effectiveness and hemodynamic safety outcomes. Second, because this study was based on routine retrospective PACU records rather than protocolized trial assessments, detailed documentation of post-treatment shivering response, including repeated severity assessments and the exact timing of rescue medication, was not uniformly available. Therefore, the time course of shivering severity and response to the initial medication could not be reliably analyzed. In addition, the administered dose, timing, infusion duration, and decision to provide additional rescue medication for dexmedetomidine and meperidine may have varied according to clinical circumstances and routine PACU practice, potentially leading to variability in outcome classification. Finally, because events of bradycardia and PONV were infrequent, statistical power may have been limited, and non-significant findings should be interpreted cautiously rather than as evidence of no difference between groups.

## Conclusion

In conclusion, among patients who developed PAS in the PACU, dexmedetomidine was associated with a significantly lower treatment failure rate than meperidine, suggesting that it may be an effective alternative when opioid sparing is desired. When used with appropriate hemodynamic monitoring, dexmedetomidine may represent an effective and safe treatment option for PAS in the PACU.

## Supplementary Information

Supplementary Material 1. Table 1. Clinical outcomes in the unmatched cohort (n = 484); Table 2. Primary and sensitivity analyses across analytic approaches

## Data Availability

The datasets used and/or analysed during the current study are available from the corresponding author on reasonable request.

## Abbreviations

PAS: Postanesthetic Shivering
PACU: Post-anesthesia Care Unit
ASA: American Society of Anesthesiologists
PONV: Postoperative Nausea and Vomiting
OR: Odds Ratio
CI: Confidence Interval
RD: Risk Difference
MD: Mean Difference
SMD: Standardized Mean Difference
