# Supplementary material for: Comparative effectiveness of dexmedetomidine and meperidine for the treatment of postanesthetic shivering: a propensity score-matched retrospective cohort study
Source: BMC Anesthesiol. 2026 Jun 26;26:556. doi: 10.1186/s12871-026-04047-9 (PMC13563817; doi:10.1186/s12871-026-04047-9)
Supplement: Supplementary file 1 [file 12871_2026_4047_MOESM1_ESM.docx]

**Supplementary Table 1.** Clinical outcomes in the unmatched cohort (n = 484)

|  | Meperidine  (n = 335) | Dexmedetomidine  (n = 149) | Effect size (95% CI) | p-value |
| --- | --- | --- | --- | --- |
| Primary outcome |  |  |  |  |
| Treatment failure* | 30 (9.0) | 5 (3.4) | OR 0.35 (0.13, 0.93) | 0.035 |
| Secondary outcomes |  |  |  |  |
| Hypotension | 12 (3.6) | 10 (6.7) | OR 1.94 (0.82, 4.59) | 0.133 |
| Bradycardia | 19 (5.7) | 8 (5.4) | OR 0.94 (0.40, 2.21) | 0.894 |
| PONV | 19 (5.7) | 2 (1.3) | OR 0.23 (0.05, 0.98) | 0.048 |
| Respiratory depression | 0 (0.0) | 0 (0.0) | Not estimable | - |
| Stay in PACU (min) | 28.19 ± 12.06 | 26.88 ± 11.40 | MD –1.31 (–3.60, 0.97) | 0.261 |
| Fentanyl dose (µg) | 59.40 ± 35.00 | 66.44 ± 34.85 | MD 7.04 (0.29, 13.79) | 0.041 |

Values are presented as number of patients (%) or mean ± standard deviation. Treatment failure was defined as the need for additional rescue anti-shivering medication in the post-anesthesia care unit after initial administration of the study drug. The effect size for respiratory depression was not estimated because no events occurred in either group. PONV: postoperative nausea and vomiting, PACU: post-anesthesia care unit.

**Supplementary Table 2.** Primary and sensitivity analyses across analytic approaches

|  | PSM  (caliper 0.2; 149 pairs) | PSM  (caliper 0.1; 144 pairs) | Unmatched full cohort multivariable adjustment  (n = 484) |
| --- | --- | --- | --- |
| Treatment failure | OR 0.33 (0.12, 0.92), p = 0.033 | OR 0.33 (0.12, 0.92),  p = 0.033 | OR 0.35 (0.13, 0.95),  p = 0.040 |
| Hypotension | OR 5.00 (1.10, 22.82),  p = 0.038 | OR 4.50 (0.97, 20.83),  p = 0.054 | OR 2.18 (0.90, 5.29),  p = 0.085 |
| Bradycardia | OR 1.60 (0.52, 4.89),  p = 0.410 | OR 1.60 (0.52, 4.89),  p = 0.410 | OR 0.96 (0.40, 2.28),  p = 0.926 |
| PONV | OR 0.67 (0.11, 3.99)*,*  p = 0.657 | OR 0.67 (0.11, 3.99),  p = 0.657 | OR 0.21 (0.05, 0.92),  p = 0.038 |
| Stay in PACU (min) | MD −2.25 (−4.99, 0.50), p = 0.108 | MD −2.67 (−5.45, 0.11),  p = 0.060 | MD −1.40 (−3.70, 0.89),  p = 0.231 |
| Fentanyl dose (µg) | MD 6.88 (−0.87, 14.62), p = 0.082 | MD 7.47 (−0.47, 15.40),  p = 0.065 | MD 7.59 (1.12, 14.05),  p = 0.022 |

Effect estimates are presented as OR or MD with 95% confidence intervals and P values for the primary propensity score-matched analysis and the two sensitivity analyses. Unmatched crude analysis is presented in Supplementary Table 1. Multivariable regression models in the unmatched full cohort included the baseline covariates used for propensity score estimation. Respiratory depression was not included in sensitivity analyses because no events occurred in either group. OR: odds ratio, MD: mean difference, PSM: propensity score matching, PONV: postoperative nausea and vomiting, PACU: post-anesthesia care unit.
